# Supplementary figures and images for: Transcriptome-wide high-throughput mapping of protein–RNA occupancy profiles using POP-seq
Source: Sci Rep. 2021 Jan 13;11:1175. doi: 10.1038/s41598-020-80846-5 (PMC7806670; doi:10.1038/s41598-020-80846-5)

NPOP-seq

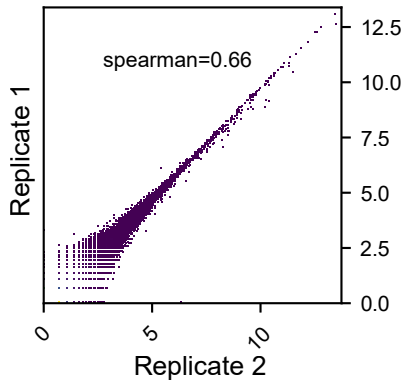

FPOP-seq

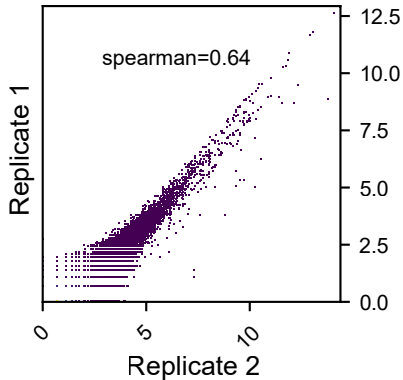

UPOP-seq

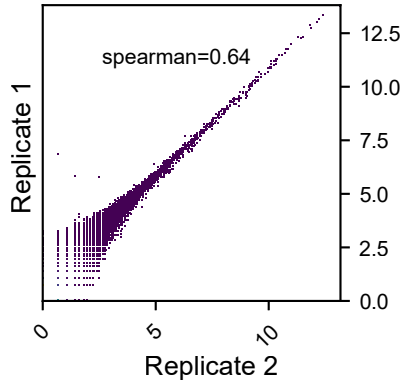

Supplement: Supplementary file 2 — Supplementary Figure S1. [file 41598_2020_80846_MOESM2_ESM.pdf]

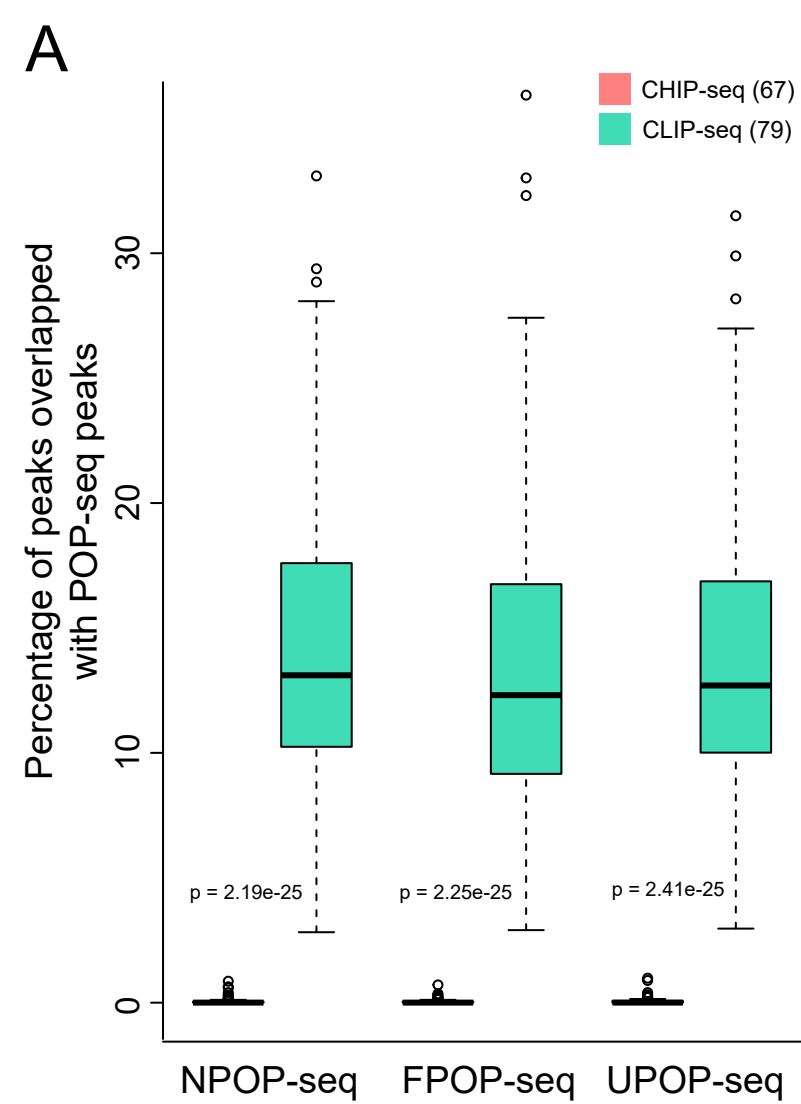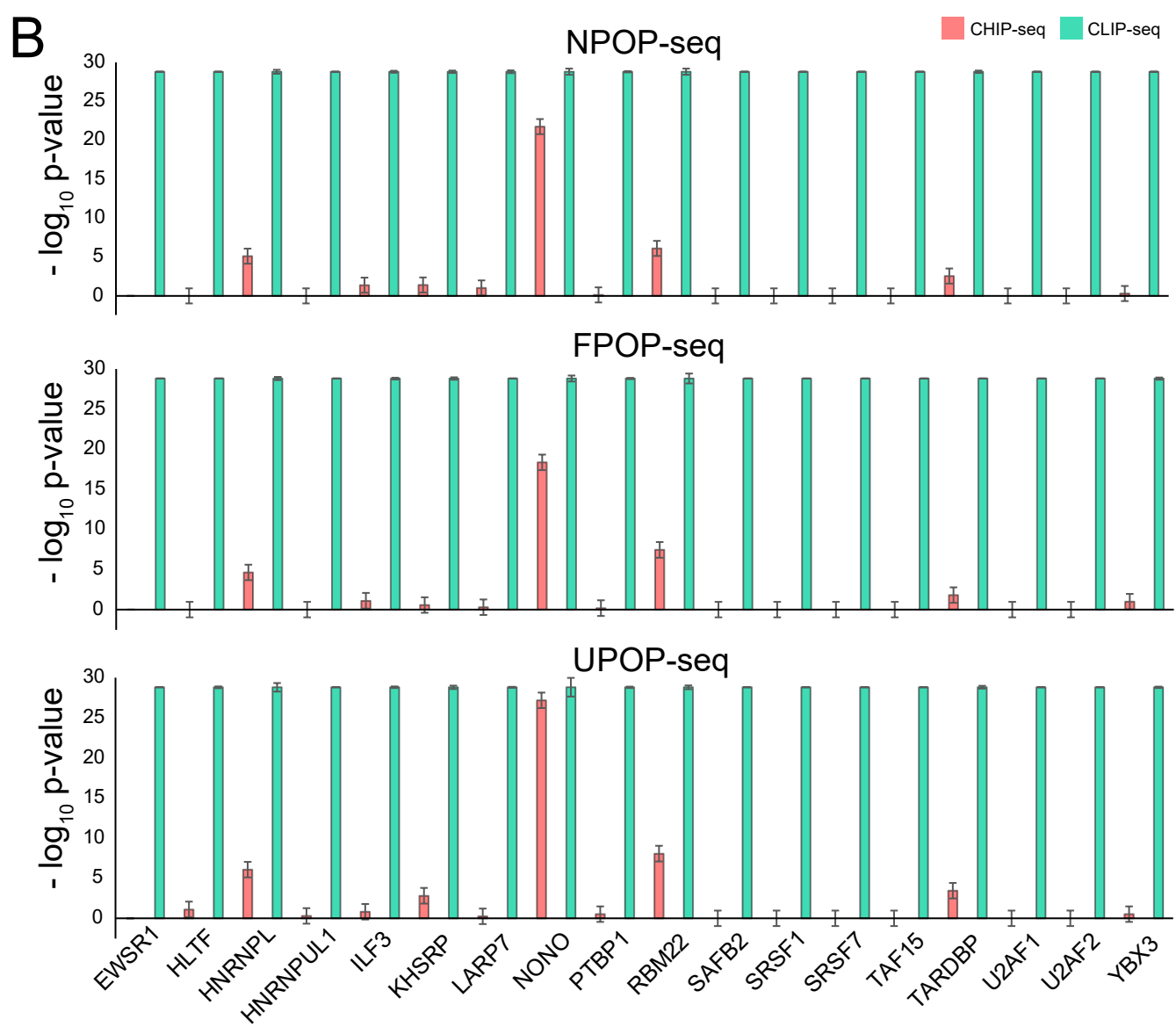

Supplement: Supplementary file 3 — Supplementary Figure S2. [file 41598_2020_80846_MOESM3_ESM.pdf]

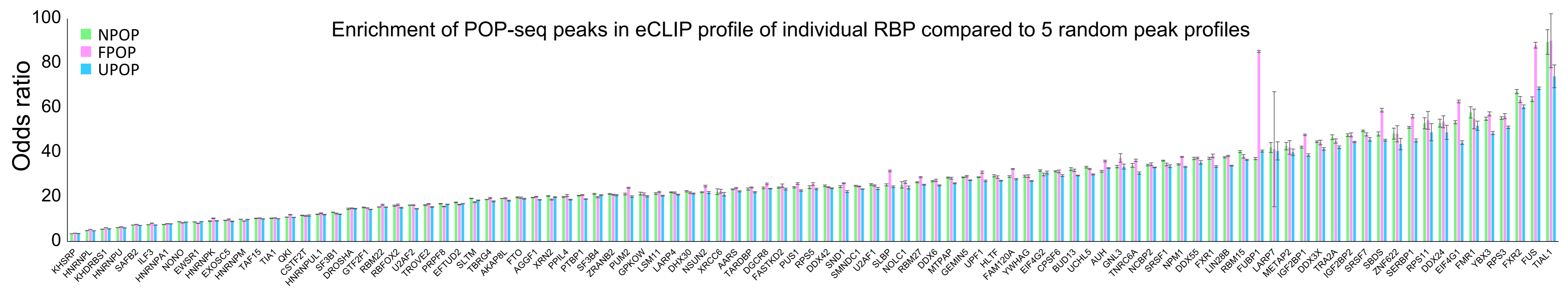

Supplement: Supplementary file 4 — Supplementary Figure S3. [file 41598_2020_80846_MOESM4_ESM.pdf]

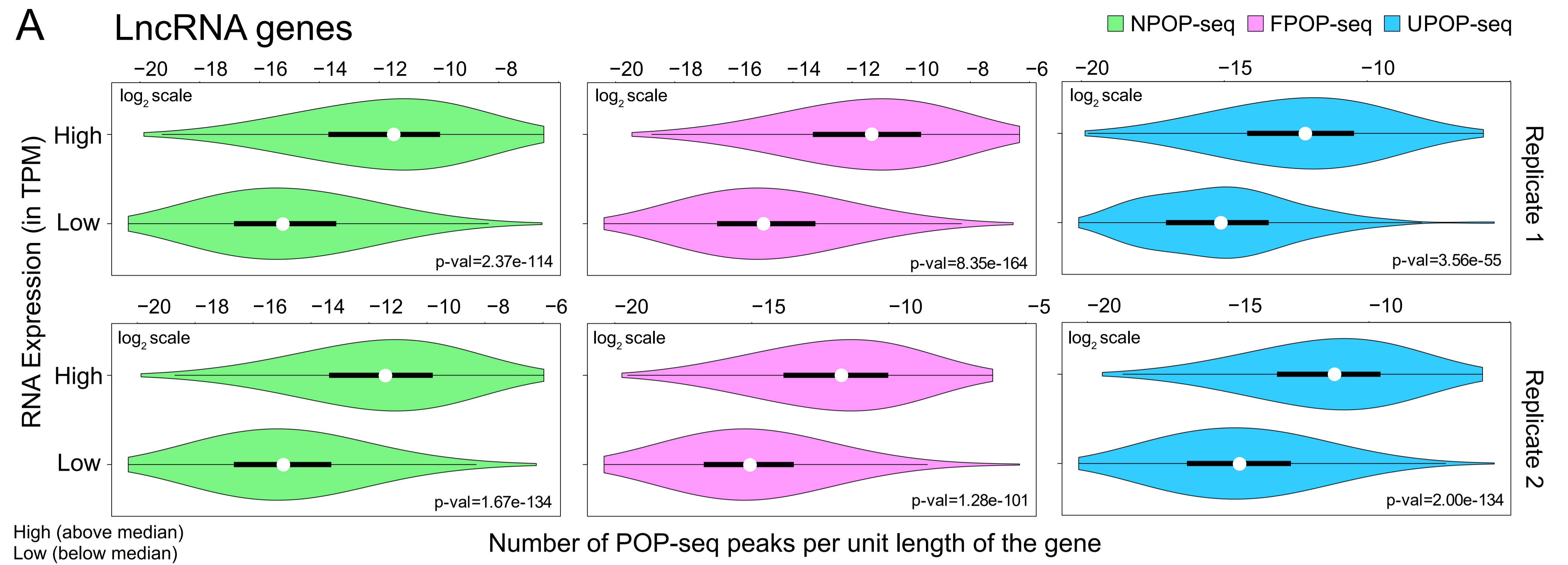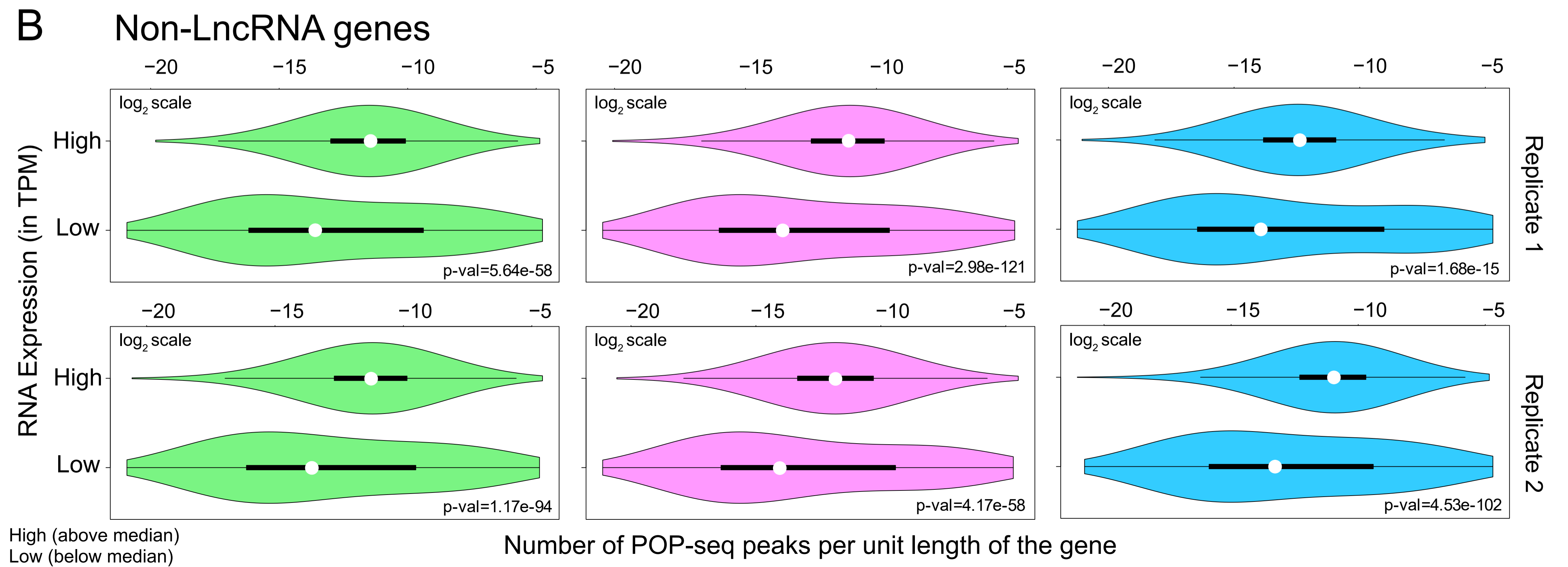

Supplement: Supplementary file 5 — Supplementary Figure S4. [file 41598_2020_80846_MOESM5_ESM.pdf]

RP11-301G19.1 (ENSG00000227706)

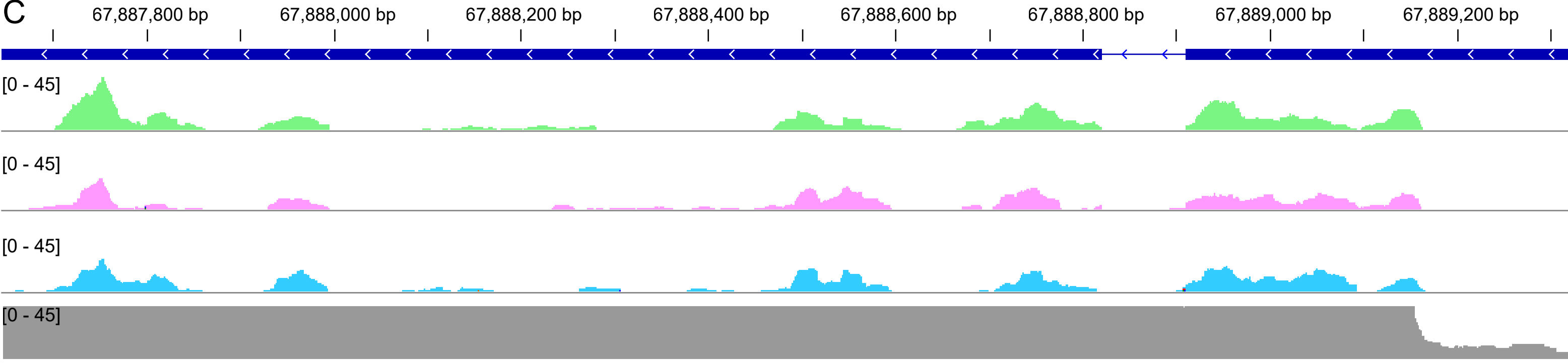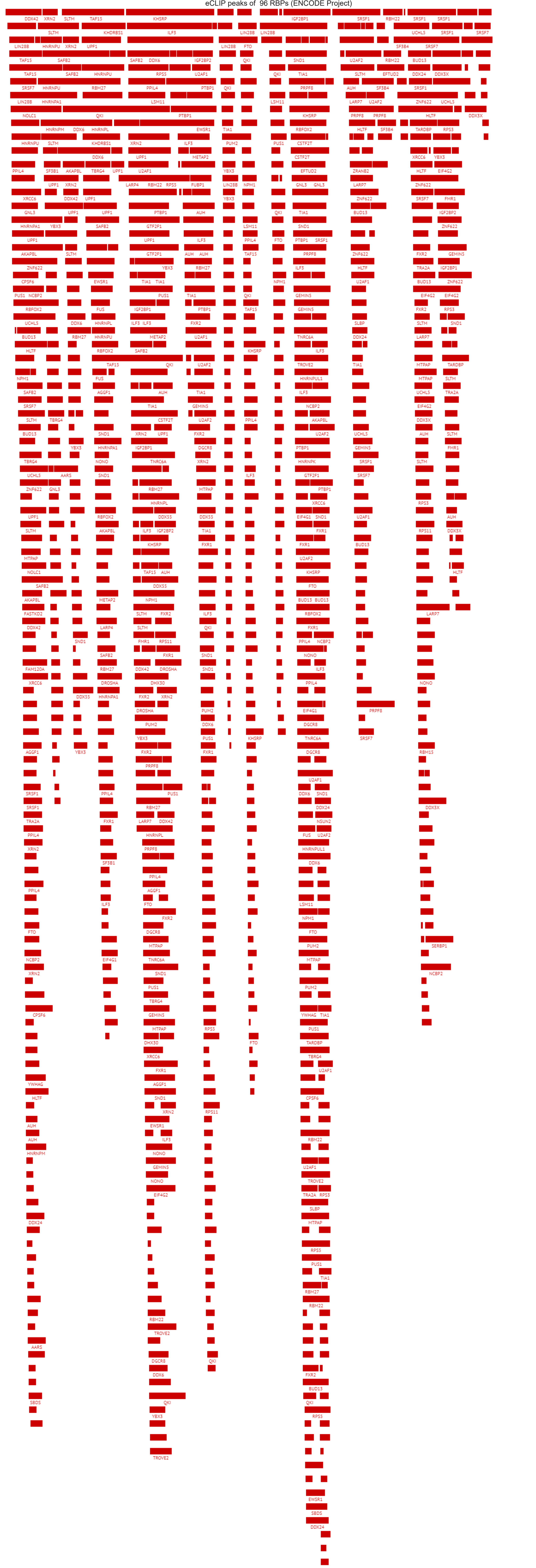

Supplement: Supplementary file 6 — Supplementary Figure S5. [file 41598_2020_80846_MOESM6_ESM.pdf]

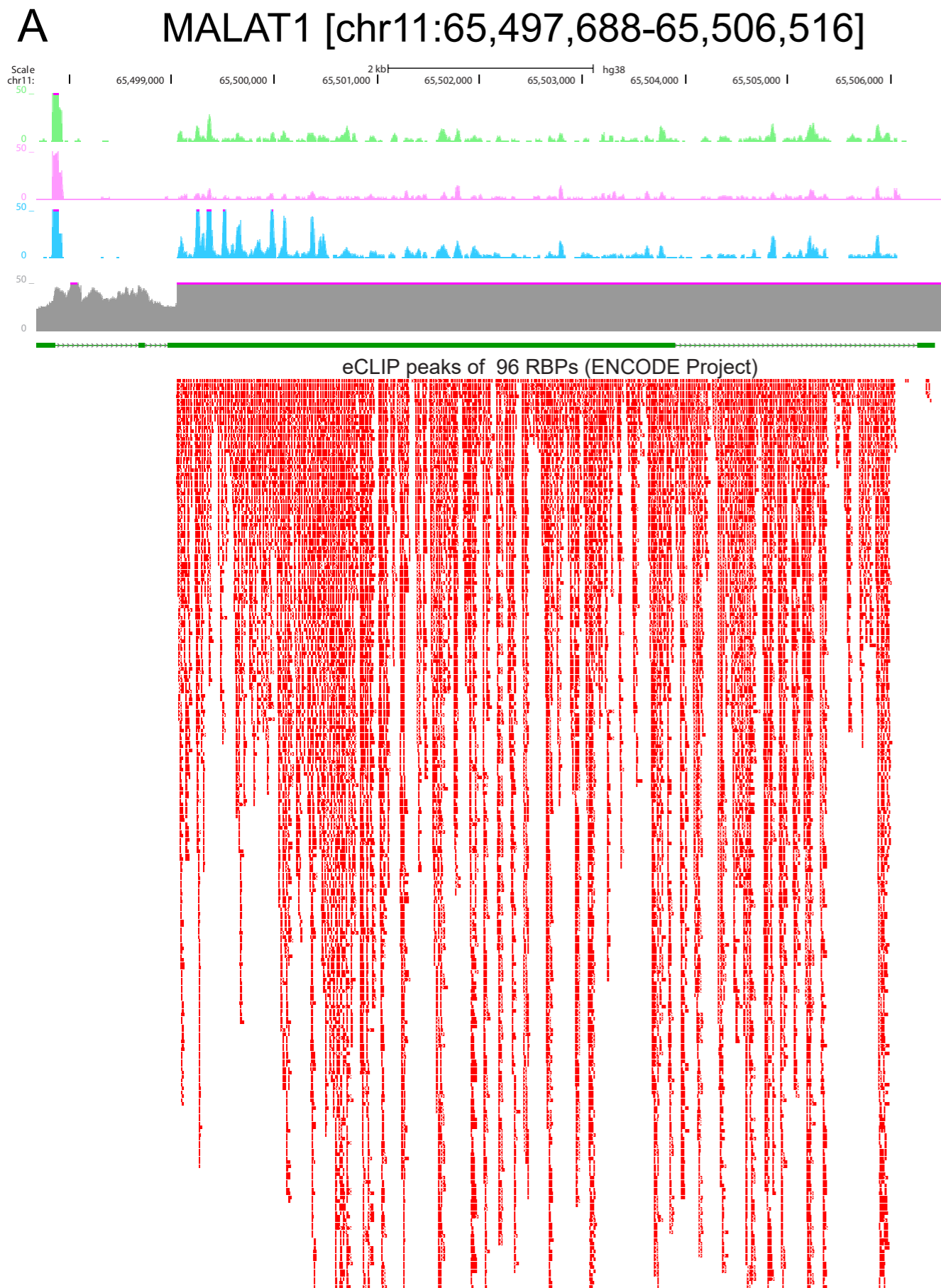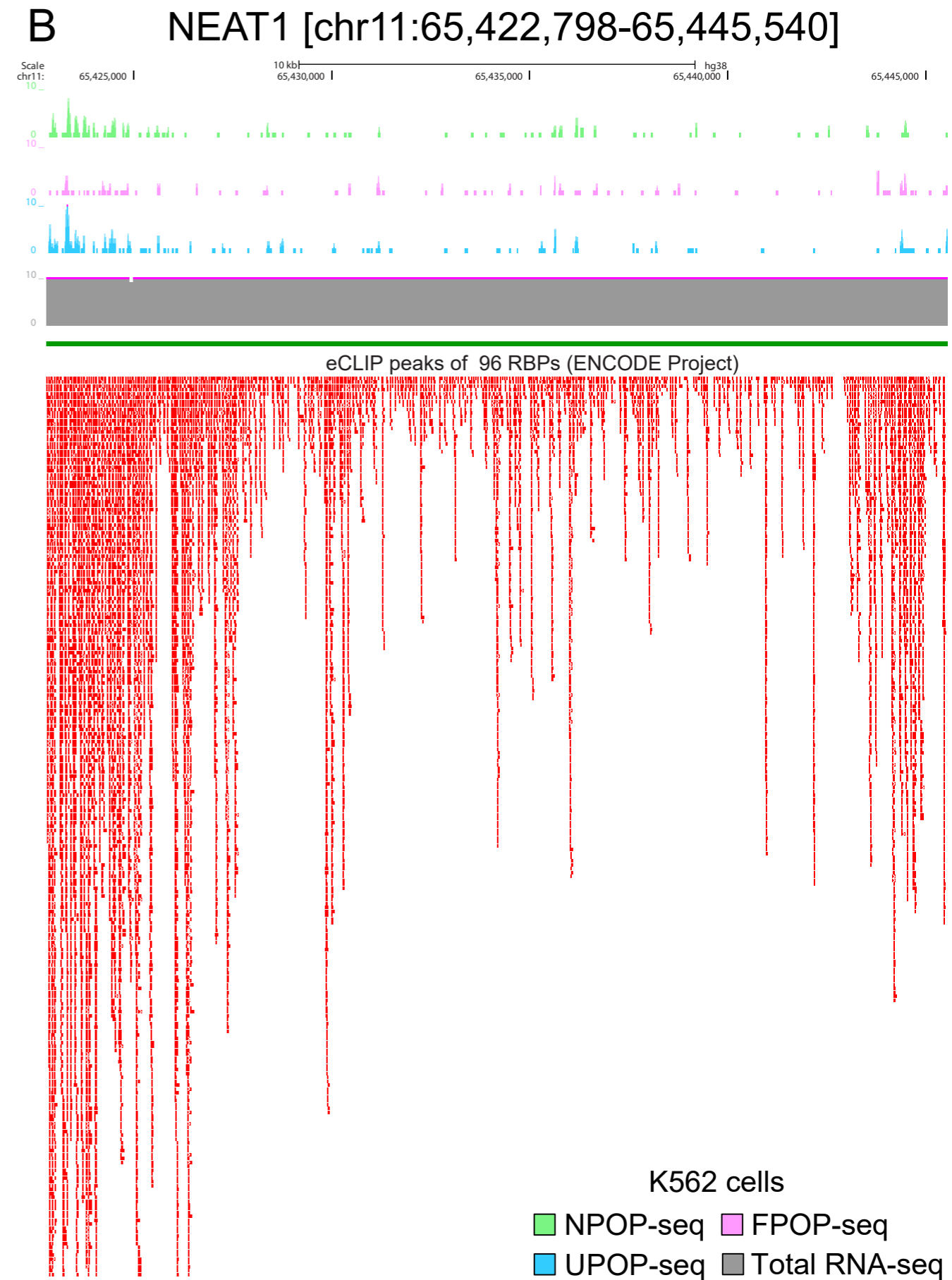

Supplement: Supplementary file 7 — Supplementary Figure S6. [file 41598_2020_80846_MOESM7_ESM.pdf]
